# Clinical, Nutritional, and Functional Evaluation of Chia Seed-Fortified Muffins

**DOI:** 10.3390/molecules27185907

**Published:** 2022-09-11

**Authors:** Roshina Rabail, Muhammad Tauseef Sultan, Abdur Rauf Khalid, Aqiba Tus Sahar, Sania Zia, Przemysław Łukasz Kowalczewski, Paweł Jeżowski, Muhammad Asim Shabbir, Rana Muhammad Aadil

**Affiliations:** 1 National Institute of Food Science and Technology, University of Agriculture, Faisalabad 38000, Pakistan; 2 Institute of Food Science and Nutrition, Bahauddin Zakariya University, Multan 60800, Pakistan; 3 Department of Livestock and Poultry Production, Faculty of Veterinary Sciences, Bahauddin Zakariya University, Multan 60800, Pakistan; 4 Department of Food Technology of Plant Origin, Poznań University of Life Sciences, 60-624 Poznań, Poland; 5 Institute of Chemistry and Technical Electrochemistry, Poznań University of Technology, 60-965 Poznań, Poland

**Keywords:** *Salvia hispanica* L., hyperlipidemia, cardiovascular diseases, fortification, cardioprotective food

## Abstract

Health-protective functional foods are gaining popularity in the world of nutrition because they promote excellent health while decreasing pharmaceutical burdens. Chia seeds (CS) (*Salvia hispanica* L.), the greatest vegetative source of α-linolenic acid, bioactive proteins, and fibers, are among the top unconventional oilseeds shown to have bounteous benefits against various non-communicable diseases. Purposely, this study was designed to integrate roasted CS powder into white-flour-based ordinary bakery goods to improve their nutritional and nutraceutical profiles. CS efficacy in normal and hyperlipidemic Sprague-Dawley rats resulted in mitigating blood glucose, triglycerides, total cholesterol, and low-density lipoprotein cholesterol while elevating high-density lipoprotein cholesterol, hematocrit, hemoglobin, red blood cell counts, and platelets. The nutritional profiling of chia-fortified muffins indicated significant increases of 47% in fat, 92% in fiber, 15% in protein, and 62% in minerals. The farinographic experiments of CS-blends revealed generally improved dough quality features with a significant rise in the degree of softening as fortification levels increased. A marketable recipe for CSF-muffins with several degrees of fortification demonstrated a significant rise in fat, 92% rise in fiber, 15% rise in protein, and 62% rise in minerals. Sensorial evaluation by trained taste panelists revealed a maximum appraisal of the 15% chia-fortified muffins due to aroma, appearance, and overall acceptability, and were forwarded for being acceptable for commercialization.

## 1. Introduction

Due to the increased prevalence of non-communicable diseases (NCDs), especially cardiovascular disorders (CVDs), the adaptation of preventive measures was kept a top-priority goal by the global NCDs action plan for 2013–2020 [1]. The global report for 2019 stated that CVDs were responsible for roughly 18.6 million deaths worldwide, with an age-adjusted incidence rate of 6431.6 per 100,000 people. While ischemic heart disease (IHD) prevalence was seen in 197.2 million people, with a higher prevalence of 113.7 in males than 83.6 in females, on the other hand, raised values of low-density lipoprotein cholesterol (LDL-C) were responsible for 4.4 million global deaths. Eastern Europe and Central Asia had remained more associated with such CVD-related mortality incidences [2]. According to future projections, around 40.5% of the US population is expected to have some type of CVD by 2030 from the ignorance of the prevailing risk factors, especially unhealthy dietary selections [3]. Such a scenario is more frequently prevalent in lower- and middle-income countries [4]. Prolonged hyperlipidemia or persistent dyslipidemia usually ends up in atherosclerosis, which is the root cause of CVD-related manifestations [5]. CVDs are one of the leading causes of disease burden in low- and middle-income countries; thus, interventions are vital to reaching both health and economic needs. Quality data on cost-effective management approaches for various chronic diseases are desperately needed. As a result, establishing heart-protective measures in people’s daily dietary modifications is critical [6]. Similar is the case with obesity, which is among the most serious public health issues with steady but continual progression and is considered a worldwide pandemic with alarmingly global prevalence. Many fad diets have popular dietary patterns known to be a quick fix for obesity and are often marketed with specific claims that these may have protective effects against obesity and certain chronic diseases such as CVDs, but still, these lack scientific evidence [7]. Hence, reverting to natural dietary resources for finding solutions could serve as a better way out.

Since ancient times, herbal plants have been used to treat and cure a variety of health-related issues. Ayurvedic medicine is widely used since it has few recorded adverse effects and a plethora of benefits [8]. A higher consumption of plant-based foods has been associated with a reduction in the risk of developing a variety of chronic diseases due to the presence of various functional nutrients in them [9]. Incorporating these plant-based items (vegetables, fruits, herbs, and spices) into one’s diet may enhance overall health and reduce cancer incidence [10]. The rising desire for foods with health-related functional benefits has sparked interest in the fortification of already existing traditional foods to enhance nutritional, health promotional, and preventive outcomes. Fatty acids are among the most popular functional meals due to their nutritional benefits. Therefore, a significant effort has been undertaken to increase the daily intake of ω-3 polyunsaturated fatty acids (PUFA) [11]. Consuming a single-vegetable oil cannot fulfill the necessary dietary requirements of ω3 PUFAs [12]. Hence, ω3 PUFAs, vitamin A, vitamin C, and a variety of phytochemicals such as polyphenols and carotenoids-rich plant-based foods that can fulfill dietary and nutritional needs along with serving functional potentials of anti-inflammatory, antioxidant, and immune protection should be added into the regular diet [13]. Especially plant-based proteins derived from nuts, seeds, and legumes have even been found to improve health outcomes in COVID-19 patients as well [14]. Hence, the incorporation of such health-protective natural foods into the contemporary dietary lifestyle can help bring salvation from many NCDs.

Bakery products are some of the most popular foods in our contemporary dietary lifestyle. The phrase “bakery items” refers to a wide variety of eatables manufactured using flour (often wheat or white flour). Cakes, muffins, bread, and cookies are likewise baked products that are rich in fats and sugars and make a tasty treat [15,16]. Bakery products are widely consumed and are quite easy for fortification at both household and industry levels. Therefore, the seeds of the chia plant (*Salvia* *hispanica* L.) being the richest source of α-linolenic acid (ALA), primary vegetative ω3 PUFA [17,18], loads of other bioactive components, and good-quality vegetative proteins and biopeptides [19], were selected for fortification into an ordinary bakery flour and to investigate the clinical, nutritional, and functional parameters of chia-fortified baked muffins.

The chia plant has gained popularity all around the world, particularly for its seeds. It is an annual herbaceous plant that is a member of the Salvia genus, Lamiales order, Lamiaceae family, and Nepetoideae subfamily, which has piqued the interest of people all over the world. The term “chia” means “oily” and comes from “Chien” or “Chian”, a Spanish word. While all of its leaves, flowers, and seeds can be used, its elegantly marbled grey to dark brown seed has been acknowledged for its medicinal advantages against a number of metabolic illnesses. Chia seeds (CS) have been developed as plant-based nutraceuticals, capturing the interest of nutritionists because of their balanced nutritional components of proteins, fiber, ω3 PUFA, vitamins, and minerals. Their healthy fat concentrations vary from 31 to 35%, with alpha-linolenic acid (ALA), i.e., ω3 PUFA content, ranging from 59 to 60%, and a cardioprotective ratio of ω6/ω3 of 1:3. CS are claimed to be one of the best sources of high-quality vegetal proteins (18.9–23%), with a plethora of peptides with bioactive potential as antioxidants, anti-diabetic, anti-inflammatory, and hypotensive properties. A dietary fiber content of 35.5% has been observed, with a ratio of insoluble to soluble dietary fiber of 4.3 and an ash content of 4.5% [19]. ALA may be transformed into various types of ω3 PUFAs such as eicosapentenoic acid (EPA) [20]. This characteristic makes its daily usage more suited for decreasing the concerning load of around 18 million fatalities, which account for one-third of all CVD-related deaths worldwide [2].

The American Heart Association recommends a minimum daily consumption of 1.4 g of ω3 PUFAs from dietary ALA sources [21] or 0.6 to 1.2% of kcal received from ALA [22]. There is indeed a space that has to be filled with practical insights for the use of CS in routine dietary patterns to attain their nutraceutical, functional, and therapeutic advantages. Therefore, in this study, the clinical efficacy of these seeds was analyzed in a rodent model using Sprague-Dawley rats. Further, these seeds were evaluated for their nutritional proximate composition and were fortified into ordinary wheat flour to develop a muffin recipe following rheological potential and sensory studies.

## 2. Results

### 2.1. Results of Clinical Study

The mean results of the physiological and laboratory biomarkers from the safety (S) and treatment (T) studies are presented in Table 1. The physiological markers investigated include food efficacy ratio (FER), total body weight increase, daily body weight gain, organ weights, and percent organ fats recorded during the experiment. CS consumption resulted in less body weight gain in both studies, but the total body weight gain values remained non-significant in both studies. On the other hand, a decrease in daily weight gain was highly significant in the T-study. There was a slight but non-significant increase in daily food intake during both studies. The FERs for the chia groups in both studies were lowered when compared to the control groups, which might be due to the lowered weight gain and increased feed intake in the chia groups. The decrease in the FER of the CS group in the T-study was very highly significant. Among organ weights, heart and spleen weights showed a slight non-significant decrease in the chia groups, whereas liver and kidney weights revealed a non-significant increase. The results for fats associated with these organs revealed lowered fat deposition in chia groups. This decrease was highly significant for fats surrounding the liver and kidney in the safety study, whereas this decline was very highly significant for fats around the heart, liver, and spleen in treatment studies.

The results of the laboratory biomarkers of the S-study in Table 2 revealed that CS consumption from D1 to D32 resulted in significantly increased levels of hematocrit (HCT), hemoglobin (Hb), red blood cells (RBCs), total leukocyte counts (TLCs), and platelet counts and significantly decreased alanine transaminase (ALT), total cholesterol (TC), triglycerides (TG), and low-density lipoprotein (LDL) cholesterol levels in the S-study. The percentage increase in healthy rats was reported as 14.40% in HCT, 23.12% in Hb, 25.67% in RBCs, 22.16% in TLCs, 30.79% in platelets, and 1.58% in high-density lipoprotein (HDL) cholesterol. On the other hand, percentage decrease from D1 to D32 in healthy rats during the S-study reported a decrease of 10.9% in ALT, 4.92% in TG, 8.08% in TC, 15.03% in LDL cholesterol, and 1.06% in glucose. The comparison between the control and chia group on D32 (identified in red font in Table 2) revealed a highly significant increase in HCT, Hb, RBCs, TLCs, and platelets; a significant increase in HDL; a highly significant decrease in glucose, TG, and TC; and a significant decrease in LDL cholesterol.

Likewise, the results of the T-study on hyperlipidemic rats are elaborated upon in Table 2. The comparative results between D1 to D32 indicated a significant increase in HCT, platelets, creatinine, and ALTs and a significant decrease in glucose, TG, TC, and LDL cholesterol. The percentage increase was reported as 8.04% for HCT, 8.80% for Hb, and 2.90% for RBCs, but these values were not significant and lower than the control group values. A percentage decrease of 8.10% in glucose, 12.40% in TG, 16.40% in TC, and 26.50% in LDL cholesterol was observed from D1 to D32 during the T-study. The results for comparison between the control and chia groups on D32 revealed highly significant amelioration for glucose, TG, TC, and LDL and significant amelioration for HDL. On the other hand, the reduction in TLCs, along with increased values for ALT and creatinine, is still uncertain.

### 2.2. Results for Proximate Nutritional Composition

The mean percentage results and the sum of squares for the proximate composition of CS, chia-seed-fortified (CSF) blends, and CSF muffins are given in Table 3. The results indicate that CS are rich in protein, fiber, fats, and mineral contents. Finely ground CS were added into plain wheat flour at fortification levels of 0, 5, 10, 15, and 20%. The resultant CSF-blends and -muffins were examined for their proximate composition and were evaluated for rheological attributes. The increasing fortification levels of CS in CSF-blends and -muffins revealed a significant decrease in their moisture contents along with a significant increase in protein content, while a highly significant increase in ash, fat, fiber, and NFE was reported.

An improved nutritional profile in CSF-blends has served as a baseline to develop innovative recipes with improved functional and nutraceutical properties. Purposely, a recipe for CSF-muffins at various fortification levels was developed and analyzed. The increasing fortification levels of muffins showed a better nutritional composition of minerals, healthy fats, good-quality vegetative proteins, and dietary fiber.

### 2.3. Rheological Studies

**Figure 1:**
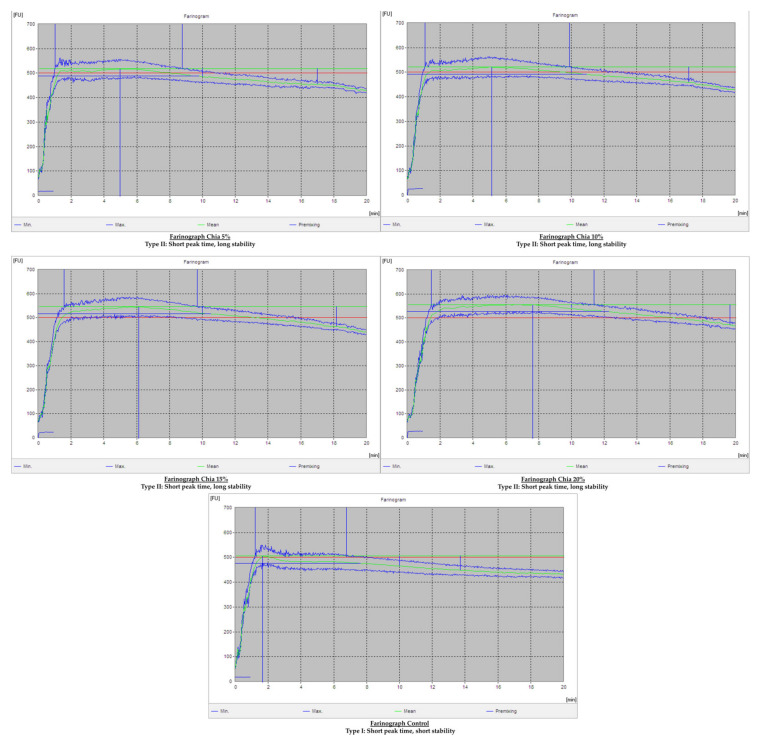
Farinographs of control and chia-supplemented flours.

Farinograph curves and their types for control, C1, C5, C10, and C20, have been elaborated upon in Figure 1 and the detailed results for rheological characteristics conducted on a farinograph have been tabulated in Table 4. These results indicate a highly significant increase in dough development time (DDT), dough stability time (DST), degree of softening (DoS), farinograph quality number (FQN), and mixing tolerance index (MTI). Changes in the results of water absorption (WA) and dough consistency (DC) remain non-significant.

### 2.4. Color Experiments

The color of the muffins has a considerable influence on their perceived acceptability. The L *, a *, and b * values for CSF-muffins are shown in Table 5. The intensity of redness was indicated by the a * value, yellowness by the b * value, and lightness by the L * value. The results showed an increase in a darker tone with a drop in L *, a decrease in yellowness with a decrease in b *, and an increase in CS in muffins in both crust and crumb, although the -a * value for minor greenness was also seen. The chroma values of the crust declined as the quantity of chia supplementation increased, and this increase was highly significant. Similar results for chroma values have been reported by [23]. Because protein concentration is inversely related to the lightness of baked goods, higher protein percentages resulted in darker muffins [24].

### 2.5. Sensory Appraisal for CSF-Muffins

**Figure 2:**
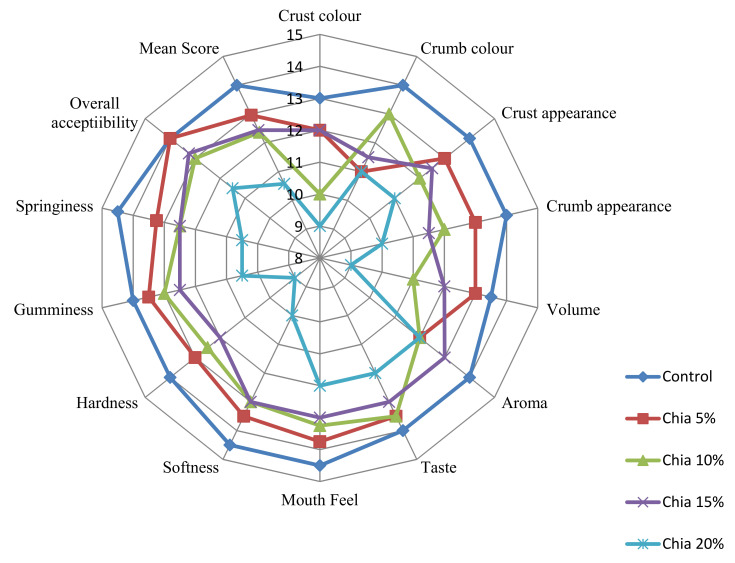
Sensorial Web for Sensory Study of CSF-Muffins.

Sensory assessment is an important stage in product development since product acceptance at any industry or even domestic level is difficult to achieve without judgment on the product’s acceptability. Figure 2 depicts how sensory rating for properties, such as crust and crumb appearance, volume, softness, hardness, springiness, and gumminess, declined as supplementing amount increased. Because increasing levels of supplementation had a detrimental influence on some sensory features in the consumer’s opinion, lower levels of supplementation (5, 10, and 15%) remained acceptable during the study.

## 3. Discussion

### 3.1. Clinical Study

The outcomes of the physical markers of the clinical study indicated improved feed consumption. Similar outcomes of enhanced energy intake and lowered body weight gain as compared to the control group were examined by Chicco et. al. [25] in a previous study, which might be linked to the improved lipid and glucose homeostasis due to the rich ALA, fiber, and minerals in chia, which could facilitate the digestion and evacuation of the bowel, resulting in improved feed consumption. Besides the increased feed intake, significantly lowered body weight gain and FER represented a better feed utilization in chia groups which might also be attributed to its rich ALA and soluble dietary fiber contents. Further, significantly lowered visceral fat depositions are also protective indicators. Therefore, chia utilization can help lower the negative pathophysiological effects caused by obesity and CVDs and can help decrease body weight gain tendencies without negatively affecting the appetite. Furthermore, these results have been supported by previous studies, as both dietary soluble fiber and ALA consumption have been associated with decreased visceral fat deposition by amelioration gene expressions [25,26,27]. Likewise, the laboratory biomarkers also revealed highly significant ameliorations in lipid profile and blood glucose levels. Similar results of lowering blood glucose, TC, TG, and LDL levels whereas increasing HDL levels have been justified in overweight adults [26,27,28,29]. Both the conversion of ALA to EPA and the greater soluble fiber content in the chia seed diet may be implicated in the decrease in hypercholesterolemia in rats [25].

The results of this study are in favor of the dietary utilization of CS not only to achieve preventive benefits but also to obtain feasibility in the treatment of hyperlipidemia. Similar findings have been supported in previous studies [26,27,28,29]. Not only the presence of ALA in CS but also the indications of highly bioactive peptides responsible for anti-diabetic, anti-hypertensive, anti-hyperlipidemic, and anti-inflammatory potentials can be the possible causative agents behind the preventive in terms of the S-study and protective in terms of the T-study [19].

### 3.2. Product Development

The results for moisture, ash, crude fat, and protein contents are in correspondence with previous studies [30,31,32,33,34]. CS fortification in plain wheat flour resulted in superior bakery flour combinations with an enhanced nutritional profile. A similar improved nutritional profile for CS-fortified wheat flour has been reported in previous studies [23,35]. On the utilization of CSF-blends, the resultant bakery items such as CSF-muffins here indicated protein-, fiber-, fat-, and mineral-rich bakery development that can not only serve beneficial against malnutrition but also could be the best choice while using to achieve its nutraceutical benefits. These results for the improved nutritional outcomes of proteins, dietary fiber, fats, and minerals have been backed up in recent studies [23,34,35,36,37,38].

The outcomes of the rheological study revealed an increase in the WA tendency of the dough. Although the increase in WA was not significant, this increase can be attributed to the increased water-holding capacity based on the gelling property of seeds and their higher soluble fiber and higher protein contents [39] and likewise to the chia mucilage’s water infinity [34]. As the increased protein and soluble fiber content in the dough resulted in increasing the WA for the full development of the dough, this might have resulted in significantly increasing the DDT and DST. Yet, the resultant dough marked a significant increase in DoS in the final dough, which might have been linked with more kneading and extra time required to adsorb more water. Flour with raised WA, MTI less than 30 B.U., and longer DDT is regarded acceptable for baking technology. Overall, the findings showed improved dough rheological qualities, with CS addition at 15% being the most acceptable based on mean DoS and DST and maximum DDT and MTI. Similar results have been obtained previously [23,40], and a similar increase in DDT, DST, and MTI in chia-supplemented flour has been reported in recent studies [34,41].

The color experiment of CSF-muffins indicated significant alterations in the chroma and hue of crust as well as chroma of crumb. These alterations represent the increasing darker tones as we increase the fortification levels and are quite closer to the chocolaty brown color that is not undesirable for many of the consumers. A similar decrease in L* in chia products has been observed in a study [34]. High L * values indicate a high light reflectivity, implying a light-color bread. The fall in L * caused by the integration of chia flour might be attributed to the color of this raw material [23].

The outcomes of the sensorial appraisal indicated that CSF-muffins remained acceptable at their 15% fortification level. However, the higher fortification levels of 15 and 20% were not rejected due to their aroma or taste but due to the increased gumminess, their acceptance beyond 15% was not favored. Similar sensorial appraisal outcomes of 5–15% chia supplementation have been reported previously [37].

## 4. Materials and Methods

During October and November 2016, the CS samples were purchased at an Al-Fateh supermarket in Faisalabad, Pakistan. All raw seed samples were kept at room temperature, and the needed CS samples were pan-roasted at a very low temperature, i.e., around 50 °C for just 3–5 min. The roasted CS samples were carefully ground in an ordinary household blender and were stored until they were used in triplicate for additional studies.

### 4.1. Clinical Study

Twenty 6–7-week-old SD rats with 187-g mean body weight were divided into two groups for clinical study: safety (S-study) and treatment (T-study). The S-study investigation attempted to evaluate whether CS intake had any harmful or protective consequences, whereas the T-study sought to identify the hypolipidemic and cardioprotective advantages of CS consumption in hyperlipidemic rats fed a diet containing 2% cholesterol for three weeks. There were two groups of five rats total in each trial. The Animal Institute of Nutrition standard standards were followed, including maintaining a temperature of 23 ± 2 °C, a 12-h cycle of light and darkness, and accurately labeled, one sq. ft metal mesh cages with water and food accessible ad libitum. The study was carried out in accordance with the National Research Council (1996) criteria in Washington, DC, USA [42], and it was authorized by the internal animal care committee of the Institute of Food Science and Nutrition, Bahauddin Zakariyya University, Pakistan (approval number IFSN/HND/21/1860).

To acclimatize to their new surroundings, the rats were fed a standard feed for one week. Following that, 32 days of experimental diets (Table 6) were given. The CS diets were created by modifying the control diet plan so that the control and CS diets were iso-caloric, as indicated in Table 6. CS were introduced to the diet in such a way that they delivered virtually identical amounts of 1 g = 1 percent of daily total calories from ALA, as recommended by the World Health Organization (WHO). About 5.4 g of ground CSF was added to the feed to make 100 g of the total feed, providing about 1 g of ALA per 100-g total weight (as it has been elaborated upon earlier in CS composition that 59–65% of CS oil is ALA). According to the WHO and the European Food Safety Authority (EFSA), 1 g per 100 g will make up 1% of the daily total calories from ALA [43,44,45].

Food consumption was tracked daily, and body weight changes were tracked weekly. At the commencement of both studies, blood samples were acquired through cardiac puncture, and rats were subsequently dissected to gather critical organs (liver, heart, kidneys, and spleen). Fat deposition and organ weight were measured in g per 100 g. Daily body weight per day and FER were calculated, as well as laboratory tests for TG, TC, LDL, HDL, AST, ALT, HCT, complete blood count (CBC), and serum creatinine. These laboratory tests were performed at the University Diagnostic Lab, Department of Veterinary Sciences, Bahauddin Zakariyya University, Multan, using commercial kits (Merck, Germany) [46]. Results were analyzed in triplicates to calculate mean values and ± standard deviations, while percentile increase or decrease was computed with the formula given below, where ˄ = percentile increase; ˅ = percentile decrease; using Omni percentile increase web calculator [16].

$$Effect=\frac{D32-D1}{D1}\;\times \;100$$

### 4.2. Proximate Nutrition Analysis

The Association of Official Analytical Chemists’ (AOAC^®^) Official Methods^SM^ [47] techniques were used to determine the proximate composition of CS in triplicates, including moisture content by Method No. 925. 08; ash content by Method No. 923.03; crude protein by Method No. 979.09; crude fat by Method No. 920.39; and crude fiber content by Method No. 962.09. The variation in total carbohydrate quantities was computed for all samples [16].

$$\mathrm{Carbohydrates}\;\left[\%\right]=100-\;\mathrm{moisture}\;\left[\%\right]-\mathrm{protein}\;\left[\%\right]-\mathrm{fat}\;\left[\%\right]-\mathrm{ash}\;\left[\%\right]$$

### 4.3. Rheological Studies of CSF-Blends on Farinograph

The CSF-blends (0, 5, 10, 15, and 20%) were made by consistently blending the finely powdered CS on a dry basis in triplicates with white wheat flour at 0, 5, 10, 15, and 20% fortification levels. The nutritional components of moisture, ash, carbohydrates, protein, fiber, and fat of CS were replaced by the nutrient contents of white wheat flour to determine the proximate composition of a specific blend. As in previous studies, these blends were rheologically investigated using a Brabender Farinograph (Electronic T150, Ohgduisburg, Germany) in line with the AACC, 2021: Method No. 54-21 procedure [48], as performed by [16,49].

### 4.4. CSF-Muffin Production

CSF-blends (0, 5, 10, 15, and 20%) were employed in a muffin recipe that may be eaten at breakfast or snack times. Table 7 lists the ingredients in a common recipe. CSF-muffins were created by substituting CSF-blends for white flour. CSF-muffins were developed in triplicates the same way as they were developed in the previous part of this study [16]. Freshly prepared CSF-muffins were cooled down at room temperature and placed into the properly labeled zip lock bags. On the next day, these freshly prepared muffins were oven-dried into powdered form and were chemically analyzed within one week.

### 4.5. CSF-Muffin Experiments

The proximate composition of CSF-muffins (0, 5, 10, 15, and 20%) was conducted in triplicates following AOAC procedures. The color experiment was performed in the Ayub Agricultural Research Institute, Faisalabad, using a CIELAB space colorimeter. The values of chroma and hue were calculated as performed in these studies [16,50].

The sensorial appraisal of CSF-muffins by the trained sensory panel was carried out on the “15th Centimeter Scale Sensorial Performa” that has been provided as a Supplementary Materials File S1. Crust color and appearance, crumb color and appearance, aroma, mouth feel, texture, taste, volume, and overall acceptance are among the ten sensory aspects evaluated. The particular characteristic steadily increases in intensity from 0 to 15. Panelists were offered plain cold water for mouth rinsing between samples and were assigned to different booths with fluorescent white lighting [16].

### 4.6. Statistical Design

Using SPSS-16 (IBM, Chicago, IL, USA), the independent sample *t*-test, one-way ANOVA, and Duncan’s multiple range test were used to compare means at the 95% (*p* < 0.05) confidence interval level.

## 5. Conclusions

The overall results of this study were quite conclusive in terms of the improved nutritional profile, especially for increasing dietary fiber, ash, and high-quality vegetative protein and fats in CS-supplemented wheat flour and its bakery product CSF-muffins. Moreover, its daily consumption in both the S- and T-studies revealed positive amelioration by lowering body weight gain, body visceral fat accumulation, TLCs, blood glucose, TG, TC, and LDL cholesterol while improving FER, HCT, Hb, platelets, and HDL cholesterol. Hence, the daily consumption of such innovative eatables can help lower the global burden of diseases and nutritional deficiencies. Chia is an outstanding source of vegetative ω3 PUFA (ALA), vegetative proteins composed of very highly bioactive peptides, rich dietary fibers, iron, and calcium contents, along with its highly significant disease-preventing and health-promoting outcomes in efficacy studies. It should be consumed on daily basis to attain these nutritional and nutraceutical benefits from it. Furthermore, the rheological properties of CS-supplemented flour blends supported their superior baking quality. The sensory evaluation suggested that the 15% supplementation dose was most acceptable. To summarize, CS can be an effective tool for improving the nutritional properties of a variety of dietary items. Based on the current research, important recommendations for the future might include creating new, revised recipes and investigating the health advantages they provide for a variety of clinical issues.

## Figures and Tables

**Table 1:** Efficacy results (mean ± SD) for physiological parameters of safety and treatment studies.

Physiological Parameters	S-Study Control	S-Study CSF	T-Study Control	T-Study CSF
Total wt. gain (g)	34.75 ± 4.031	32.15 ± 1.738 ns	55.87 ± 3.679	51.00 ± 1.460 ns
Wt. gain (g)/day	1.24 ± 0.144	1.24 ± 0.244 ns	1.73 ± 0.136	1.62 ± 0.029 ***
Food intake (g)/day	25 ± 1.174	26.95 ± 1.369 ns	27.63 ± 2.576	28.57 ± 0.719 ns
FER%	4.95 ± 0.667	4.23 ± 0.669 ns	6.26 ± 0.220	5.63 ± 0.898 ***
Heart wt. (g)	0.40 ± 0.027	0.38 ± 0.037 ns	0.35 ± 0.029	0.32 ± 0.024 ns
Liver wt. (g)	3.42 ± 0.191	3.26 ± 0.351 ns	2.78 ± 0.123	2.93 ± 0.063 ns
Kidney wt. (g)	0.33 ± 0.019	0.34 ± 0.029 ns	0.28 ± 0.025	0.31 ± 0.025 ns
Spleen wt. (g)	0.19 ± 0.008	0.16 ± 0.037 ns	0.16 ± 0.021	0.14 ± 0.021 ns
Heart fat%	9.07 ± 0.739	7.80 ± 0.685 *	12.40 ± 0.561	10.5 ± 0.021 ***
Liver fat%	9.39 ± 0.455	5.30 ± 0.886 ***	4.39 ± 0.103	2.40 ± 0.241 ***
Kidney fat%	32.00 ± 0.483	27.93 ± 5.678 **	34.30 ± 1.213	34.30 ± 1.415 ns
Spleen fat%	43.925 ± 10.274	32.80 ± 1.029 ns	22.00 ± 0.794	15.00 ± 0.406 ***

* Mean/% ± SD; FER—food efficacy ratio = (Wt. gain/day ÷ food intake/day) × 100; S = safety study, T = treatment study. *** = very highly significant at *p* < 0.001; ** = highly significant at *p* < 0.01; * = significant at *p* < 0.05; ns = non-significant at *p* < 0.05.

**Table 2:** Efficacy Results (mean ± SD) for Laboratory biomarkers of S- and T-Study.

Groups		S-Study						T-Study					
		S-Study Control			S-Study CSF			T-Study Control			T-Study CSF		
	Labs	D-1	D-32	Effect	D-1	D-32	Effect	D-1	D-32	Effect	D-1	D-32	Effect
HCT (%)		34.23 ± 0.75	35.67 ± 0.52 ns	4.20 ˄	36.65 ± 0.45	41.93 ± 0.45 ******	14.40 ˄	30.77 ± 0.37	40.33 ± 0.60 ***	31.07 ˄	37.30 ± 0.785	40.30 ± 1.143 ** ns	8.04 ˄
Hb (g/dL)		11.55 ± 0.42	13.65 ± 0.73 **	18.18 ˄	12.63 ± 0.58	15.55 ± 0.48 *****	23.12 ˄	11.53 ± 0.59	14.43 ± 0.42 ***	25.15 ˄	13.26 ± 0.890	14.43 ± 0.524 ns ns	8.80 ˄
RBCs (10^6^/µL)		5.80 ± 0.20	6.52 ± 0.30 **	12.41 ˄	6.04 ± 0.21	7.34 ± 0.23 *****	25.68 ˄	9.62 ± 0.42	7.32 ± 0.24 ***	23.91 ˅	6.88 ± 0.131	7.08 ± 1.14 ns ns	2.90 ˄
TLCs (X 10^3^/µL)		8.73 ± 0.35	7.37 ± 0.51 **	15.57 ˅	10.60 ± 0.73	12.95 ± 0.67 *****	22.16 ˄	18.26 ± 0.25	12.63 ± 0.35 ***	30.83 ˅	13.46 ± 0.294	11.63 ± 0.325 *****	13.50 ˅
Platelets (10^3^/µL)		561 ± 2.95	469 ± 1.93 **	16.39 ˅	591 ± 2.25	773 ± 2.52 ******	30.79 ˄	480 ± 4.27	694 ± 4.92 ***	44.58 ˄	381 ± 1.643	700 ± 2.124 *** ns	90.70 ˄
MCV (fL)		57.90 ± 0.84	54.77 ± 0.31 ***	5.40 ˅	61.00 ± 1.56	57.20 ± 0.77 *****	6.22 ˅	52.36 ± 0.92	55.13 ± 1.29 **	5.29 ˄	54.10 ± 0.477	55.73 ± 1.577 ns ns	3.00 ˄
MCH (pg)		19.87 ± 0.36	20.97 ± 0.90 ns	5.54 ˄	34.23 ± 0.99	36.80 ± 0.57 *****	7.5 ˄	32.10 ± 0.75	19.76 ± 0.29 ***	38.44 ˅	19.23 ± 0.777	20.10 ± 1.047 ns ns	4.50 ˄
MCHC (g/dL)		34.37 ± 0.82	38.28 ± 0.62 ***	11.37 ˄	34.23 ± 0.71	36.80 ± 0.45 *****	7.5 ˄	30.13 ± 1.27	35.80 ± 0.32 ***	18.8 ˄	35.5 3 ± 0.737	36.10 ± 0.217 ns ns	1.60 ˄
Glucose (mg/dL)		83.46 ± 1.78	93.25 ± 2.68 ***	11.73 ˄	83.46 ± 1.78	82.57 ± 0.83 ns ***	1.06 ˅	92.82 ± 0.25	96.73 ± 0.75 ***	4.21 ˄	92.82 ± 0.258	85.24 ± 0.258 ******	8.10 ˅
AST (U/L)		66.96 ± 0.55	70.55 ± 1.43 **	5.36 ˄	66.96 ± 0.55	70.32 ± 0.40 *** ns	5.02 ˄	112.77 ± 2.41	108.98 ± 1.23 ns	3.36 ˅	112.77 ± 2.418	103.73 ± 2.790 ***	8.00 ˅
ALT (U/L)		43.29 ± 0.72	40.61 ± 1.45 **	6.19 ˅	43.29 ± 0.72	38.57 ± 0.72 *** ns	10.90 ˅	33.29 ± 0.54	48.25 ± 0.54 ***	44.93 ˄	33.29 ± 0.546	49.53 ± 1.047 *** ns	48.70 ˄
Creatinine (U/L)		0.30 ± 0.01	0.35 ± 0.03 ns	16.67 ˄	0.30 ± 0.01	0.45 ± 0.03 *****	50 ˄	0.30 ± 0.02	0.30 ± 0.01 ns	0	0.30 ± 0.020	0.49 ± 0.026 ******	63.00 ˄
TG (mg/dL)		91.35 ± 1.08	92.90 ± 0.23 ns	1.69 ˄	91.35 ± 1.08	86.85 ± 0.79 ******	4.92 ˅	206.27 ± 1.88	209.03 ± 1.88 ns	1.33 ˄	206.27 ± 1.885	180.66 ± 1.534 ******	12.40 ˅
TC (mg/dL)		93.28 ± 1.27	90.24 ± 1.24 *	3.33 ˅	93.28 ± 1.27	86.67 ± 0.63 *****	8.08 ˅	206.5 ± 2.33	232.70 ± 1.12 ***	12.73 ˄	206.5 ± 2.332	172.58 ± 8.462 ******	16.40 ˅
HDL (mg/dL)		33.50 ± 0.58	31.54 ± 1.55 ns	5.85 ˅	33.50 ± 0.58	34.03 ± 0.53 ns *	1.58 ˄	45.38 ± 2.05	44.17 ± 1.04 ns	2.67 ˅	45.38 ± 2.056	48.35 ± 1.691 ns *	6.50 ˄
VLDL (mg/dL)		18.27 ± 0.25	18.58 ± 0.45 ns	1.69 ˄	18.27 ± 0.25	17.37 ± 0.55 ns ***	4.92 ˅	41.25 ± 0.65	41.81 ± 0.46 ns	1.35 ˄	41.25 ± 0.653	36.13 ± 1.079 *****	12.40 ˅
LDL (mg/dL)		41.51 ± 0.69	40.12 ± 0.54 ns	3.34 ˅	41.51 ± 0.69	35.27 ± 0.49 ****	15.03 ˅	119.87 ± 1.09	146.72 ± 1.37 ***	22.39 ˄	119.87 ± 1.093	88.10 ± 1.743 ******	26.50 ˅

S = safety, T = treatment, hemoglobin (Hb), red blood cells (RBCs), total leukocyte counts (TLCs), hematocrit concentration (HCT), mean corpuscular volume (MCV), mean corpuscular hemoglobin (MCH), mean corpuscular hemoglobin concentration (MCHC), alanine transaminase (ALT), aspartate transaminase (AST), total cholesterol (TC), triglycerides (TG), high-density lipoproteins (HDL), low-density lipoproteins (LDL). * D—days; effect = [(D32-D1)÷D1]×100; ˄—percentage increase; ˅—percentage decrease. *** = very highly significant at *p* < 0.001; ** = highly significant at *p* < 0.01; * = significant at *p* < 0.05; ns = non-significant at *p* < 0.05 on comparison D1 to D32. Red font *** = very highly significant at *p* < 0.001; ** = highly significant at *p* < 0.01; * = significant at *p* < 0.05; and ns = non-significant at *p* < 0.05 on comparison D32 of control group to D32 of CS groups.

**Table 3:** Proximate Composition (mean ± SD) and Sum of Squares of CS, their Blends, and Muffins.

Seed		Moisture	Ash	Crude Fat	Crude Protein	Crude Fiber	NFE
CS%		5.43 ± 0.21	4.26 ± 0.02	37.33 ± 1.36	20.34 ± 1.65	16.88 ± 1.04	15.75 ± 1.29
CSF-blends%	C0	12.250 ^a^	0.312 ^e^	1.143 ^e^	11.500 ^c^	1.320 ^e^	73.475 ^a^
	C5	11.909 ^ab^	0.510 ^d^	2.953 ^d^	11.942 ^bc^	2.098 ^d^	70.589 ^ab^
	C10	11.568 ^abc^	0.707 ^c^	4.762 ^c^	12.384 ^abc^	2.876 ^c^	67.703 ^bc^
	C15	11.278 ^bc^	0.852 ^b^	6.597 ^b^	12.850 ^ab^	3.391 ^b^	65.033 ^cd^
	C20	10.987 ^c^	0.998 ^a^	8.431 ^a^	13.316 ^a^	3.906 ^a^	62.363 ^d^
CSF-muffins%	C0	34.830 ± 1.066 ^a^	1.430 ± 0.031 ^d^	11.730 ± 0.528 ^d^	9.860 ± 0.339 ^c^	1.200 ± 0.060 ^e^	40.950 ± 2.341 ^a^
	C5	34.130 ± 1.533 ^ab^	1.900 ± 0.080 ^c^	14.360 ± 0.442 ^c^	10.180 ± 0.450 ^bc^	1.450 ± 0.086 ^d^	37.980 ± 1.390 ^ab^
	C10	32.670 ± 1.266 ^abc^	2.170 ± 0.083 ^b^	15.670 ± 0.258 ^b^	10.470 ± 0.382 ^bc^	1.730 ± 0.074 ^c^	37.290 ± 1.036 ^b^
	C15	31.850 ± 1.293 ^bc^	2.230 ± 0.037 ^ab^	16.630 ± 0.649 ^a^	10.830 ± 0.565 ^ab^	2.010 ± 0.067 ^b^	36.450 ± 0.794 ^b^
	C20	31.550 ± 0.785 ^c^	2.330 ± 0.068 ^a^	17.230 ± 0.621 ^a^	11.420 ± 0.329 ^a^	2.310 ± 0.046 ^a^	35.160 ± 2.717 ^b^
Sum of squares	Blends	2.994 *	0.887 **	99.628 **	6.166 *	12.678 **	231.72 **
	Muffins	24.478 *	1.586 **	57.297 **	4.360 *	2.315 **	56.199 *

C0 = acts as control; means sharing the same letters in a column are not significantly different from each other at *p* < 0.05; ** = highly significant at *p* < 0.01; * = significant at *p* < 0.05; ns = non-significant at *p* < 0.05.

**Table 4:** Farinographic characteristics (mean ± SD) and their sum of squares for CSF-blends.

T	WA (%)	DDT (Min)	DST (Min)	DoS (FU)	DoS-ICC (FU)	DC (FU)	FQN	MTI (Min)
C0	60.000 ± 2.457 ^a^	1.700 ± 0.047 ^d^	5.600 ± 0.164 ^d^	41.000 ± 1.047 ^a^	58.000 ± 2.296 ^b^	506.000 ± 18.433 ^b^	76.000 ± 1.738 ^d^	6.700 ± 0.266 ^d^
C5	61.000 ± 1.678 ^a^	5.000 ± 0.352 ^c^	7.800 ± 0.194 ^c^	33.000 ± 1.588 ^b^	63.000 ± 2.642 ^b^	518.000 ± 19.333 ^ab^	98.000 ± 3.850 ^c^	10.000 ± 0.507 ^c^
C10	61.400 ± 1.609 ^a^	5.200 ± 0.175 ^c^	8.800 ± 0.063 ^b^	25.000 ± 0.606 ^c^	63.000 ± 4.542 ^b^	522.000 ± 27.528 ^ab^	110.000 ± 4.694 ^b^	10.200 ± 0.266 ^c^
C15	62.000 ± 1.971 ^a^	6.200 ± 0.118 ^b^	8.100 ± 0.190 ^c^	27.000 ± 1.351 ^c^	83.000 ± 3.848 ^a^	546.000 ± 25.557 ^ab^	105.000 ± 4.471 ^bc^	11.200 ± 0.195 ^b^
C20	62.800 ± 2.203 ^a^	7.700 ± 0.415 ^a^	9.900 ± 0.320 ^a^	15.000 ± 0.551 ^d^	86.000 ± 3.312 ^a^	556.000 ± 16.381 ^a^	123.000 ± 4.581 ^a^	12.700 ± 0.841 ^a^
Sum of squares	13.296 ns	58.572 **	30.156 **	1118.160 **	1996.160 **	5146.128 ns	3615.124 **	58.545 **

WA = water absorption; DDT = dough development time; DST = dough stability time; DoS = degree of softening DoS-ICC = degree of softening-ICC; DC = dough consistency; FQN = farinograph quality number; MTI = mixing tolerance index. C0 = acts as control; means sharing the same letters in a column are not significantly different from each other at *p* < 0.05. ** = highly significant at *p* < 0.01; ns = non-significant at *p* < 0.05.

**Table 5:** Color indices (mean ± SD) and their sum of squares of CSF-muffins.

CSF-Muffins	Supplementation Level %	L *	a *	b *	Chroma	Hue
Crust	C0	53.39 ± 1.82 ^a^	2.15 ± 0.09 ^b^	19.99 ± 0.64 ^b^	20.11 ± 0.63 ^b^	1.46 ± 0.04 ^a^
	C5	51.51 ± 1.88 ^ab^	−1.01 ± 0.03 ^c^	14.77 ± 0.95 ^c^	14.80 ± 0.46 ^c^	−1.50 ± 0.10 ^b^
	C10	49.24 ± 1.15 ^b^	−1.16 ± 0.02 ^d^	12.62 ± 0.59 ^d^	12.67 ± 0.32 ^d^	−1.48 ± 0.05 ^b^
	C15	34.23 ± 0.71 ^d^	5.00 ± 0.08 ^a^	24.26 ± 0.93 ^a^	24.77 ± 0.66 ^a^	1.37 ± 0.04 ^a^
	C20	44.60 ± 2.32 ^c^	−1.55 ± 0.03 ^e^	9.60 ± 0.36 ^e^	9.72 ± 0.43 ^e^	−1.41 ± 0.04 ^b^
Crumb	C0	64.78 ± 2.19 ^a^	−4.99 ± 0.14 ^e^	19.76 ± 0.84 ^a^	20.38 ± 0.89 ^a^	−1.32 ± 0.04 ^a^
	C5	60.59 ± 2.34 ^b^	−4.34 ± 0.13 ^d^	19.86 ± 0.85 ^a^	20.33 ± 0.79 ^a^	−1.36 ± 0.03 ^a^
	C10	50.42 ± 2.27 ^c^	−3.07 ± 0.16 ^c^	11.88 ± 0.23 ^b^	12.27 ± 0.53 ^b^	−1.32 ± 0.05 ^a^
	C15	47.97 ± 1.06 ^c^	−1.81 ± 0.07 ^a^	9.86 ± 0.66 ^c^	10.02 ± 0.29 ^c^	−1.39 ± 0.05 ^a^
	C20	47.06 ± 1.05 ^c^	−2.19 ± 0.09 ^b^	10.15 ± 0.33 ^c^	10.38 ± 0.31 ^c^	−1.36 ± 0.04 ^a^
Sum of squares	Crust	702.673 **	96.199 **	413.07 ***	434.357 **	29.835 **
	Crumb	770.330 **	22.322 **	310.338 **	330.966 **	0.010 ns

C0 = acts as control; means sharing the same letters in a column are not significantly different from each other at *p* < 0.05. *** = very highly significant at *p* < 0.001; ** = highly significant at *p* < 0.01; ns = non-significant at *p* < 0.05.

**Table 6:** Diet for the control, cholesterol, and CS-fed rats.

Dietary Components	Control		Cholesterol		CS	
	g	Kcal	g	Kcal	g	Kcal
Corn starch	65	260	65	260	64.48	257.92
Casein	12	48	12	48	10.92	43.68
Corn oil	10	90	8	72	8	72
Cholesterol	-	-	2	18	-	-
Cellulose	5.25	-	5.25	-	4.50	-
Wheat bran	5.25	-	5.25	-	4.50	-
Mineral mix.	1.5	-	1.5	-	1.5	-
Vitamin mix.	1	-	1	-	1	-
CSF	-	-	-	-	5.4	24.40
Total	100	398	100	398	100.3	398

**Table 7:** List of ingredients (g) for standard/control recipe of muffins.

Ingredients	Quantity per Batch (Makes 6)	Quantity per Muffin
Flour	100	16.66
Baking powder	5	0.83
Baking soda	1.25	0.21
Sugar	50	8.33
Cinnamon powder	1.25	0.83
Vanilla essence	1	0.16
Yogurt	60	10
Milk	30	5
Lemon juice	2	0.33
Coconut oil	20	3.33
Egg whites	30	5
Net weight	300	50

## Data Availability

All data generated or analyzed during this study are included in this published article.

## Supplementary Materials

The following supporting information can be downloaded at: https://www.mdpi.com/article/10.3390/molecules27185907/s1. The questionnaire for sensory appraisal is provided in the supplementary files.

## References

[1] World Health Organization (2013). Follow-Up to the Political Declaration of the High-Level Meeting of the General Assembly on the Prevention and Control of Non-Communicable Diseases.

[2] American Heart Association (2021). 2021 Heart Disease & Stroke Statistical Update Fact Sheet Global Burden of Disease.

[3] Heidenreich P.A., Trogdon J.G., Khavjou O.A., Butler J., Dracup K., Ezekowitz M.D., Finkelstein E.A., Hong Y., Johnston S.C., Khera A. (2011). Forecasting the Future of Cardiovascular Disease in the United States A Policy Statement from the American Heart Association. Circulation.

[4] World Heart Federation (2018). CVD Advocacy Toolkit—The Road to 2018.

[5] Bruen R., Fitzsimons S., Belton O. (2017). Atheroprotective effects of conjugated linoleic acid. Br. J. Clin. Pharm..

[6] Siscovick D.S., Barringer T.A., Fretts A.M., Wu J.H.Y., Lichtenstein A.H., Costello R.B., Kris-Etherton P.M., Jacobson T.A., Engler M.B., Alger H.M. (2017). Omega-3 Polyunsaturated Fatty Acid (Fish Oil) Supplementation and the Prevention of Clinical Cardiovascular Disease: A Science Advisory from the American Heart Association. Circulation.

[7] Tahreem A., Rakha A., Rabail R., Nazir A., Socol C.T. (2022). Fad Diets: Facts and Fiction. Front. Nutr..

[8] Khan M.I., Maqsood M., Saeed R.A., Alam A., Sahar A., Kieliszek M., Miecznikowski A., Muzammil H.S., Aadil R.M. (2021). Phytochemistry, food application, and therapeutic potential of the medicinal plant (Withania coagulans): A review. Molecules.

[9] Wang H., Wang J., Qiu C., Ye Y., Guo X., Chen G., Li T., Wang Y., Fu X., Liu R.H. (2017). Comparison of phytochemical profiles and health benefits in fiber and oil flaxseeds (*Linum usitatissimum* L.). Food Chem..

[10] Saeed R.A., Maqsood M., Saeed R.A., Shehzad H., Khan M.I., Asghar L., Nisa S.U., Aadil R.M. (2022). Plant-based foods and hepatocellular carcinoma: A review on mechanistic understanding. Crit. Rev. Food Sci. Nutr..

[11] Konieczka P., Czauderna M., Smulikowska S. (2017). The enrichment of chicken meat with omega-3 fatty acids by dietary fish oil or its mixture with rapeseed or flaxseed—Effect of feeding duration: Dietary fish oil, flaxseed, and rapeseed and n-3 enriched broiler meat. Anim. Feed Sci. Technol..

[12] Rabail R., Shabbir M.A., Sahar A., Miecznikowski A., Kieliszek M., Aadil R.M. (2021). An intricate review on nutritional and analytical profiling of coconut, flaxseed, olive, and sunflower oil blends. Molecules.

[13] Shabbir M.A., Mehak F., Khan Z.M., Ahmed W., Haq S.M.A.U., Khan M.R., Bhat Z.F., Aadil R.M. (2022). Delving the role of nutritional psychiatry to mitigate the COVID-19 pandemic induced stress, anxiety and depression. Trends Food Sci. Technol..

[14] Rabail R., Saleem J., Tanveer Z., Patching S.G., Khalid A.R., Sultan M.T., Manzoor M.F., Karrar E., Inam-Ur-Raheem M., Shabbir M.A. (2021). Nutritional and lifestyle changes required for minimizing the recovery period in home quarantined COVID-19 patients of Punjab, Pakistan. Food Sci. Nutr..

[15] Martinez M.M., Gomez M. (2019). Current trends in the realm of baking: When indulgent consumers demand healthy sustainable foods. Foods.

[16] Rabail R., Shabbir M.A., Ahmed W., Inam-Ur-Raheem M., Khalid A.R., Sultan M.T., Aadil R.M. (2022). Nutritional, functional, and therapeutic assessment of muffins fortified with garden cress seeds. J. Food Process. Preserv..

[17] Ciftci O.N., Przybylski R., Rudzińska M. (2012). Lipid components of flax, perilla, and chia seeds. Eur. J. Lipid Sci. Technol..

[18] Spotorno V., Mateo C.M., Diehl B.W.K., Nolasco S.M., Toma M.C. (2011). Characterization of chia seed oils obtained by pressing and solvent extraction. J. Food Compos. Anal..

[19] Rabail R., Khan M.R., Mehwish H.M., Rajoka M.S.R., Lorenzo J.M., Kieliszek M., Khalid A.R., Shabbir M.A., Aadil R.M. (2021). An overview of chia seed (*Salvia hispanica* L.) bioactive peptides’ derivation and utilization as an emerging nutraceutical food. Front. Biosci. Landmark 2021, 26, 643–654. Front. Biosci. Landmark.

[20] Alagawany M., Elnesr S.S., Farag M.R., El-Sabrout K., Alqaisi O., Dawood M.A.O., Soomro H., Abdelnour S.A. (2020). Nutritional significance and health benefits of omega-3, -6 and -9 fatty acids in animals. Anim. Biotechnol..

[21] Gowda A., Sharma V., Goyal A., Singh A.K., Arora S. (2018). Process optimization and oxidative stability of omega-3 ice cream fortified with flaxseed oil microcapsules. J. Food Sci. Technol..

[22] McGuire S., U.S. Department of Agriculture, U.S. Department of Health and Human Services (2011). Dietary Guidelines for Americans, 2010.

[23] Guiotto E.N., Tomas M.C., Haros C.M. (2020). Development of Highly Nutritional Breads with By-Products of Chia (*Salvia hispanica* L.) Seeds. Foods.

[24] Fagundes G.A., Rocha M., Salas-Mellado M.M. (2018). Improvement of protein content and effect on technological properties of wheat bread with the addition by cobia (Rachycentron canadum). Food Res..

[25] Chicco A.G., D’Alessandro M.E., Hein G.J., Oliva M.E., Lombardo Y.B. (2009). Dietary chia seed (*Salvia hispanica* L.) rich in a-linolenic acid improves adiposity and normalises hypertriacylglycerolaemia and insulin resistance in dyslipaemic rats. Br. J. Nutr..

[26] Nieman D.C., Cayea E.J., Austin M.D., Henson D.A., McAnulty S.R., Jin F. (2009). Chia seed does not promote weight loss or alter disease risk factors in overweight adults. Nutr. Res..

[27] Nieman D.C., Gillitt N., Jin F., Henson D.A., Kennerly K., Shanely R.A., Ore B., Su M., Schwartz S. (2012). Chia seed supplementation and disease risk factors in overweight women: A metabolomics investigation. J. Altern. Complement. Med..

[28] Alamri E. (2019). The Influence of Two Types of Chia Seed on Some Physiological Parameters in Diabetic Rats. Int. J. Pharm. Res. Allied Sci..

[29] Da Silva C.S., Monteiro C.R.D.A., da Silva G.H.F., Sarni R.O.S., Souza F.I.S., Feder D., Messias M.C.F., Carvalho P.D.O., Alberici R.M., Cunha I.B.S. (2020). Assessing the Metabolic Impact of Ground Chia Seed in Overweight and Obese Prepubescent Children: Results of a Double-Blind Randomized Clinical Trial. J. Med. Food.

[30] Kulczyński B., Kobus-Cisowska J., Taczanowski M., Kmiecik D., Gramza-Michałowska A. (2019). The Chemical Composition and Nutritional Value of Chia Seeds-Current State of Knowledge. Nutrients.

[31] Mihafu F.D., Kiage B.N., Okoth J.K., Nyerere A.K. (2019). Nutritional Composition and Qualitative Phytochemical Analysis of Chia Seeds (*Salvia hispanica* L.) Grown in East Africa. Curr. Nutr. Food Sci..

[32] Sá A.G.A., da Silva D.C., Pacheco M.T.B., Moreno Y.M.F., Carciofi B.A.M. (2021). Oilseed by-products as plant-based protein sources: Amino acid profile and digestibility. Futur. Foods.

[33] Da Silva B.P., Anunciação P.C., da Silva Matyelka J.C., della Lucia C.M., Martino H.S.D., Pinheiro-Sant’Ana H.M. (2017). Chemical composition of Brazilian chia seeds grown in different places. Food Chem..

[34] Iglesias-Puig E., Haros M. (2013). Evaluation of performance of dough and bread incorporating chia (*Salvia hispanica* L.). Eur. Food Res. Technol..

[35] Hafeez A., Ahmad A., Amir R.M., Kaleem M. (2019). Quality evaluation of coconut–flaxseed balls enriched with chiaseeds. J. Food Process. Preserv..

[36] Abdullah M., Masood B. (2022). Chia Seeds as Potential Nutritional and Functional Ingredients: A Review of their Applications for Various Food Industries. J. Nutr. Food Sci. Technol..

[37] Sandri L.T.B., Santos F.G., Fratelli C., Capriles V.D. (2017). Development of gluten-free bread formulations containing whole chia flour with acceptable sensory properties. Food Sci. Nutr..

[38] Romankiewicz D., Hassoon W.H., Cacak-Pietrzak G., Sobczyk M.B., Wirkowska-Wojdyła M., Ceglińska A., Dziki D. (2017). The effect of chia seeds (*Salvia hispanica* L.) addition on quality and nutritional value of wheat bread. J. Food Qual..

[39] Toliba A., Mohamed A. (2019). The Effect of Garden Cress Seeds Addition on Rheological Properties of Wheat Flour and Chocolate Flavored Cupcake. Egypt. J. Food Sci..

[40] Zettel V., Krämer A., Hecker F., Hitzmann B. (2015). Influence of gel from ground chia (*Salvia hispanica* L.) for wheat bread production. Eur. Food Res. Technol..

[41] Hrušková M., Švec I., Jurinová I. (2013). Chemometrics of wheat composites with hemp, teff, and chia flour: Comparison of rheological features. Int. J. Food Sci..

[42] NRC (1996). Guide for the Care and Use of Laboratory Animals.

[43] Engel P. Essential Fatty Acids Intake Recommendations. https://www.nutri-facts.org/en_US/nutrients/items/essential-fatty-acids/essential-fatty-acids/intake-recommendations.html.

[44] Bresson J.L., Flynn A., Heinonen M., Hulshof K., Korhonen H., Lagiou P., Løvik M., Marchelli R., Martin A., Moseley B. (2009). Labelling reference intake values for n-3 and n-6 polyunsaturated fatty acids. Eur. Food Saf. Auth. J..

[45] Gebauer S.K., Psota T.L., Harris W.S., Kris-Etherton P.M. (2006). N-3 fatty acid dietary recommendations and food sources to achieve essentiality and cardiovascular benefits. Am. J. Clin. Nutr..

[46] Sultan M.T., Butt M.S., Ahmad R.S., Pasha I., Ahmad A.N., Qayyum M.M.N. (2012). Supplementation of Nigella sativa fixed and essential oil mediates potassium bromate induced oxidative stress and multiple organ toxicity. Pak. J. Pharm. Sci..

[47] AOAC (2019). Official Methods of Analysis of AOAC International.

[48] Ashraf W., Shehzad A., Sharif H.R., Aadil R.M., Rafiq Khan M., Zhang L. (2020). Influence of selected hydrocolloids on the rheological, functional, and textural properties of wheat-pumpkin flour bread. J. Food Process. Preserv..

[49] Rehman S., Paterson A., Hussain S. (2007). Influence of partial substitution of wheat flour with vetch (*Lathyrus sativus* L.) flour on quality characteristics of doughnuts. LWT-Food Sci. Technol..

[50] Alshehry G.A. (2019). Technological and sensory characteristics of biscuits fortified with garden cress (*Lepidum sativum*) seeds. Life Sci. J..

