# Supplementary material for: Clinical, Nutritional, and Functional Evaluation of Chia Seed-Fortified Muffins
Source: Molecules. 2022 Sep 11;27(18):5907. doi: 10.3390/molecules27185907 (PMC9503555; doi:10.3390/molecules27185907)
Supplement: Supplementary file 1 [file molecules-27-05907-s001.zip › molecules-1877653-supplementary.pdf]

SM. Fifteen Centimeter Scale Performa for Sensorial Appraisal

Time_______________ Date_______________

Instructions for "15cm Scale":

- Read carefully the description of individual attributes.
- Rate your acceptance by placing marks between **"0-15cm"** inside the appropriate column in the table given ahead.
- There is a gradual increase from 0 toward 15 in the appropriate intensity of trait, i.e.,
  - A: 0___1 ____2 ____3 ____4 ____5 ____6 ____7 ____8 ____9 ____10 ____11 ____12 ____13 ____14 ____15
    - ***0** stands for least intensity/absence of relevant trait*
    - ***15** stands for the most intensity/maximum prevalence of relevant trait*
- Don't disturb the order of the samples and the code allotted to them.

Description of individual attributes/traits:

1. **Crust colour**: Intensity of "Caramelized light Brown" colour, typical of muffin's crust.
2. **Crumb Colour**: Intensity of "Creamy light Brown" colour, typical of muffin's crumb.
3. **Crust Appearance**: Uniformed appearance of crust firmness as of typical muffins.
4. **Crumb Appearance**: Uniformed appearance of air cells (porosity) as of typical muffins.
5. **Volume:** Appropriate size/height gained that is associated with a typical muffin.
6. **Aroma**: Degree of Aroma intensity associated with typical muffins.
7. **Taste:** Intensity of perceived taste of a typical muffin bite.
8. **Mouth feel**: Appealing sensation associated with mild sweetness and freshness as associated with a typical muffin.
9. **Texture**:
   9.1 **Softness**:
   9.2 **Hardness**:
   9.3 **Gumminess**:
   9.4 **Springiness**: Intensity of desired springiness associated with a typical muffin
10. **Overall acceptability**: Overall impression of the muffin based on all attributes.

---

1. Crust colour: Intensity of “Caramelized Brown” colour, typical of muffin crust

Not at all intense Very intense

A: ______1 ______2 ______3 ______4 ______5 ______6 ______7 ______8 ______9 ______10 ______11 ______12 ______13 ______14 ______15

B: ______1 ______2 ______3 ______4 ______5 ______6 ______7 ______8 ______9 ______10 ______11 ______12 ______13 ______14 ______15

C: ______1 ______2 ______3 ______4 ______5 ______6 ______7 ______8 ______9 ______10 ______11 ______12 ______13 ______14 ______15

D: ______1 ______2 ______3 ______4 ______5 ______6 ______7 ______8 ______9 ______10 ______11 ______12 ______13 ______14 ______15

E: ______1 ______2 ______3 ______4 ______5 ______6 ______7 ______8 ______9 ______10 ______11 ______12 ______13 ______14 ______15

F: ______1 ______2 ______3 ______4 ______5 ______6 ______7 ______8 ______9 ______10 ______11 ______12 ______13 ______14 ______15

G: ______1 ______2 ______3 ______4 ______5 ______6 ______7 ______8 ______9 ______10 ______11 ______12 ______13 ______14 ______15

H: ______1 ______2 ______3 ______4 ______5 ______6 ______7 ______8 ______9 ______10 ______11 ______12 ______13 ______14 ______15

2. Crumb Colour: intensity of “Light Brown” colour, typical of muffin crumb

Not at all intense Very intense

A: ______1 ______2 ______3 ______4 ______5 ______6 ______7 ______8 ______9 ______10 ______11 ______12 ______13 ______14 ______15

B: ______1 ______2 ______3 ______4 ______5 ______6 ______7 ______8 ______9 ______10 ______11 ______12 ______13 ______14 ______15

C: ______1 ______2 ______3 ______4 ______5 ______6 ______7 ______8 ______9 ______10 ______11 ______12 ______13 ______14 ______15

D: ______1 ______2 ______3 ______4 ______5 ______6 ______7 ______8 ______9 ______10 ______11 ______12 ______13 ______14 ______15

E: ______1 ______2 ______3 ______4 ______5 ______6 ______7 ______8 ______9 ______10 ______11 ______12 ______13 ______14 ______15

F: ______1 ______2 ______3 ______4 ______5 ______6 ______7 ______8 ______9 ______10 ______11 ______12 ______13 ______14 ______15

G: ______1 ______2 ______3 ______4 ______5 ______6 ______7 ______8 ______9 ______10 ______11 ______12 ______13 ______14 ______15

H: ______1 ______2 ______3 ______4 ______5 ______6 ______7 ______8 ______9 ______10 ______11 ______12 ______13 ______14 ______15

3. Crust Appearance: the uniformed appearance of crust firmness, typical of muffins

Not at all uniform Very uniform

A: ______1 ______2 ______3 ______4 ______5 ______6 ______7 ______8 ______9 ______10 ______11 ______12 ______13 ______14 ______15

B: ______1 ______2 ______3 ______4 ______5 ______6 ______7 ______8 ______9 ______10 ______11 ______12 ______13 ______14 ______15

C: ______1 ______2 ______3 ______4 ______5 ______6 ______7 ______8 ______9 ______10 ______11 ______12 ______13 ______14 ______15

D: ______1 ______2 ______3 ______4 ______5 ______6 ______7 ______8 ______9 ______10 ______11 ______12 ______13 ______14 ______15

E: ______1 ______2 ______3 ______4 ______5 ______6 ______7 ______8 ______9 ______10 ______11 ______12 ______13 ______14 ______15

F: ______1 ______2 ______3 ______4 ______5 ______6 ______7 ______8 ______9 ______10 ______11 ______12 ______13 ______14 ______15

G: ______1 ______2 ______3 ______4 ______5 ______6 ______7 ______8 ______9 ______10 ______11 ______12 ______13 ______14 ______15

H: ______1 ______2 ______3 ______4 ______5 ______6 ______7 ______8 ______9 ______10 ______11 ______12 ______13 ______14 ______15

4. Crumb Appearance: The uniformed appearance of air cells (porosity) present in Muffin crumb

Not at all uniform Very uniform

A: ______1 ______2 ______3 ______4 ______5 ______6 ______7 ______8 ______9 ______10 ______11 ______12 ______13 ______14 ______15

B: ______1 ______2 ______3 ______4 ______5 ______6 ______7 ______8 ______9 ______10 ______11 ______12 ______13 ______14 ______15

C: ______1 ______2 ______3 ______4 ______5 ______6 ______7 ______8 ______9 ______10 ______11 ______12 ______13 ______14 ______15

D: ______1 ______2 ______3 ______4 ______5 ______6 ______7 ______8 ______9 ______10 ______11 ______12 ______13 ______14 ______15

E: ______1 ______2 ______3 ______4 ______5 ______6 ______7 ______8 ______9 ______10 ______11 ______12 ______13 ______14 ______15

F: ______1 ______2 ______3 ______4 ______5 ______6 ______7 ______8 ______9 ______10 ______11 ______12 ______13 ______14 ______15

G: ______1 ______2 ______3 ______4 ______5 ______6 ______7 ______8 ______9 ______10 ______11 ______12 ______13 ______14 ______15

H: ______1 ______2 ______3 ______4 ______5 ______6 ______7 ______8 ______9 ______10 ______11 ______12 ______13 ______14 ______15

5. Volume

Not at all gained Maximum gained

A: ______1 ______2 ______3 ______4 ______5 ______6 ______7 ______8 ______9 ______10 ______11 ______12 ______13 ______14 ______15

B: ______1 ______2 ______3 ______4 ______5 ______6 ______7 ______8 ______9 ______10 ______11 ______12 ______13 ______14 ______15

C: ______1 ______2 ______3 ______4 ______5 ______6 ______7 ______8 ______9 ______10 ______11 ______12 ______13 ______14 ______15

D: ______1 ______2 ______3 ______4 ______5 ______6 ______7 ______8 ______9 ______10 ______11 ______12 ______13 ______14 ______15

E: ______1 ______2 ______3 ______4 ______5 ______6 ______7 ______8 ______9 ______10 ______11 ______12 ______13 ______14 ______15

F: ______1 ______2 ______3 ______4 ______5 ______6 ______7 ______8 ______9 ______10 ______11 ______12 ______13 ______14 ______15

G: ______1 ______2 ______3 ______4 ______5 ______6 ______7 ______8 ______9 ______10 ______11 ______12 ______13 ______14 ______15

H: ______1 ______2 ______3 ______4 ______5 ______6 ______7 ______8 ______9 ______10 ______11 ______12 ______13 ______14 ______15

6. Aroma: Degree of Aroma intensity associated with typical muffins

Not at all intense Very intense

A: ______1 ______2 ______3 ______4 ______5 ______6 ______7 ______8 ______9 ______10 ______11 ______12 ______13 ______14 ______15

B: ______1 ______2 ______3 ______4 ______5 ______6 ______7 ______8 ______9 ______10 ______11 ______12 ______13 ______14 ______15

C: ______1 ______2 ______3 ______4 ______5 ______6 ______7 ______8 ______9 ______10 ______11 ______12 ______13 ______14 ______15

D: ______1 ______2 ______3 ______4 ______5 ______6 ______7 ______8 ______9 ______10 ______11 ______12 ______13 ______14 ______15

E: ______1 ______2 ______3 ______4 ______5 ______6 ______7 ______8 ______9 ______10 ______11 ______12 ______13 ______14 ______15

F: ______1 ______2 ______3 ______4 ______5 ______6 ______7 ______8 ______9 ______10 ______11 ______12 ______13 ______14 ______15

G: ______1 ______2 ______3 ______4 ______5 ______6 ______7 ______8 ______9 ______10 ______11 ______12 ______13 ______14 ______15

H: ______1 ______2 ______3 ______4 ______5 ______6 ______7 ______8 ______9 ______10 ______11 ______12 ______13 ______14 ______15

7. Taste: Intensity of perceived taste of a typical muffin bite

Not at all intense Very intense

A: ______1 ______2 ______3 ______4 ______5 ______6 ______7 ______8 ______9 ______10 ______11 ______12 ______13 ______14 ______15

B: ______1 ______2 ______3 ______4 ______5 ______6 ______7 ______8 ______9 ______10 ______11 ______12 ______13 ______14 ______15

C: ______1 ______2 ______3 ______4 ______5 ______6 ______7 ______8 ______9 ______10 ______11 ______12 ______13 ______14 ______15

D: ______1 ______2 ______3 ______4 ______5 ______6 ______7 ______8 ______9 ______10 ______11 ______12 ______13 ______14 ______15

E: ______1 ______2 ______3 ______4 ______5 ______6 ______7 ______8 ______9 ______10 ______11 ______12 ______13 ______14 ______15

F: ______1 ______2 ______3 ______4 ______5 ______6 ______7 ______8 ______9 ______10 ______11 ______12 ______13 ______14 ______15

G: ______1 ______2 ______3 ______4 ______5 ______6 ______7 ______8 ______9 ______10 ______11 ______12 ______13 ______14 ______15

H: ______1 ______2 ______3 ______4 ______5 ______6 ______7 ______8 ______9 ______10 ______11 ______12 ______13 ______14 ______15

8. Mouth feel:

Not at all appealing Very appealing

A: ______1 ______2 ______3 ______4 ______5 ______6 ______7 ______8 ______9 ______10 ______11 ______12 ______13 ______14 ______15

B: ______1 ______2 ______3 ______4 ______5 ______6 ______7 ______8 ______9 ______10 ______11 ______12 ______13 ______14 ______15

C: ______1 ______2 ______3 ______4 ______5 ______6 ______7 ______8 ______9 ______10 ______11 ______12 ______13 ______14 ______15

D: ______1 ______2 ______3 ______4 ______5 ______6 ______7 ______8 ______9 ______10 ______11 ______12 ______13 ______14 ______15

E: ______1 ______2 ______3 ______4 ______5 ______6 ______7 ______8 ______9 ______10 ______11 ______12 ______13 ______14 ______15

F: ______1 ______2 ______3 ______4 ______5 ______6 ______7 ______8 ______9 ______10 ______11 ______12 ______13 ______14 ______15

G: ______1 ______2 ______3 ______4 ______5 ______6 ______7 ______8 ______9 ______10 ______11 ______12 ______13 ______14 ______15

H: ______1 ______2 ______3 ______4 ______5 ______6 ______7 ______8 ______9 ______10 ______11 ______12 ______13 ______14 ______15

9. Texture:

9.5 Softness:

Not at all intense Very intense

A: ______1 ______2 ______3 ______4 ______5 ______6 ______7 ______8 ______9 ______10 ______11 ______12 ______13 ______14 ______15

B: ______1 ______2 ______3 ______4 ______5 ______6 ______7 ______8 ______9 ______10 ______11 ______12 ______13 ______14 ______15

C: ______1 ______2 ______3 ______4 ______5 ______6 ______7 ______8 ______9 ______10 ______11 ______12 ______13 ______14 ______15

D: ______1 ______2 ______3 ______4 ______5 ______6 ______7 ______8 ______9 ______10 ______11 ______12 ______13 ______14 ______15

E: ______1 ______2 ______3 ______4 ______5 ______6 ______7 ______8 ______9 ______10 ______11 ______12 ______13 ______14 ______15

F: ______1 ______2 ______3 ______4 ______5 ______6 ______7 ______8 ______9 ______10 ______11 ______12 ______13 ______14 ______15

G: ______1 ______2 ______3 ______4 ______5 ______6 ______7 ______8 ______9 ______10 ______11 ______12 ______13 ______14 ______15

H: ______1 ______2 ______3 ______4 ______5 ______6 ______7 ______8 ______9 ______10 ______11 ______12 ______13 ______14 ______15

9.6 Hardness:

Not at all intense Very intense

A: ______1 ______2 ______3 ______4 ______5 ______6 ______7 ______8 ______9 ______10 ______11 ______12 ______13 ______14 ______15

B: ______1 ______2 ______3 ______4 ______5 ______6 ______7 ______8 ______9 ______10 ______11 ______12 ______13 ______14 ______15

C: ______1 ______2 ______3 ______4 ______5 ______6 ______7 ______8 ______9 ______10 ______11 ______12 ______13 ______14 ______15

D: ______1 ______2 ______3 ______4 ______5 ______6 ______7 ______8 ______9 ______10 ______11 ______12 ______13 ______14 ______15

E: ______1 ______2 ______3 ______4 ______5 ______6 ______7 ______8 ______9 ______10 ______11 ______12 ______13 ______14 ______15

F: ______1 ______2 ______3 ______4 ______5 ______6 ______7 ______8 ______9 ______10 ______11 ______12 ______13 ______14 ______15

G: ______1 ______2 ______3 ______4 ______5 ______6 ______7 ______8 ______9 ______10 ______11 ______12 ______13 ______14 ______15

H: ______1 ______2 ______3 ______4 ______5 ______6 ______7 ______8 ______9 ______10 ______11 ______12 ______13 ______14 ______15

9.7 Gumminess:

Not at all intense Very intense

A: ______1 ______2 ______3 ______4 ______5 ______6 ______7 ______8 ______9 ______10 ______11 ______12 ______13 ______14 ______15

B: ______1 ______2 ______3 ______4 ______5 ______6 ______7 ______8 ______9 ______10 ______11 ______12 ______13 ______14 ______15

C: ______1 ______2 ______3 ______4 ______5 ______6 ______7 ______8 ______9 ______10 ______11 ______12 ______13 ______14 ______15

D: ______1 ______2 ______3 ______4 ______5 ______6 ______7 ______8 ______9 ______10 ______11 ______12 ______13 ______14 ______15

E: ______1 ______2 ______3 ______4 ______5 ______6 ______7 ______8 ______9 ______10 ______11 ______12 ______13 ______14 ______15

F: ______1 ______2 ______3 ______4 ______5 ______6 ______7 ______8 ______9 ______10 ______11 ______12 ______13 ______14 ______15

G: ______1 ______2 ______3 ______4 ______5 ______6 ______7 ______8 ______9 ______10 ______11 ______12 ______13 ______14 ______15

H: ______1 ______2 ______3 ______4 ______5 ______6 ______7 ______8 ______9 ______10 ______11 ______12 ______13 ______14 ______15

9.8 Springiness: Intensity of desired springiness associated with a typical muffin

Not at all intense | Very intense

A: ______1 ______2 ______3 ______4 ______5 ______6 ______7 ______8 ______9 ______10 ______11 ______12 ______13 ______14 ______15

B: ______1 ______2 ______3 ______4 ______5 ______6 ______7 ______8 ______9 ______10 ______11 ______12 ______13 ______14 ______15

C: ______1 ______2 ______3 ______4 ______5 ______6 ______7 ______8 ______9 ______10 ______11 ______12 ______13 ______14 ______15

D: ______1 ______2 ______3 ______4 ______5 ______6 ______7 ______8 ______9 ______10 ______11 ______12 ______13 ______14 ______15

E: ______1 ______2 ______3 ______4 ______5 ______6 ______7 ______8 ______9 ______10 ______11 ______12 ______13 ______14 ______15

F: ______1 ______2 ______3 ______4 ______5 ______6 ______7 ______8 ______9 ______10 ______11 ______12 ______13 ______14 ______15

G: ______1 ______2 ______3 ______4 ______5 ______6 ______7 ______8 ______9 ______10 ______11 ______12 ______13 ______14 ______15

H: ______1 ______2 ______3 ______4 ______5 ______6 ______7 ______8 ______9 ______10 ______11 ______12 ______13 ______14 ______15

10. Overall acceptability: Overall impression of the muffin based on all attributes

Not at all intense | Very intense

A: ______1 ______2 ______3 ______4 ______5 ______6 ______7 ______8 ______9 ______10 ______11 ______12 ______13 ______14 ______15

B: ______1 ______2 ______3 ______4 ______5 ______6 ______7 ______8 ______9 ______10 ______11 ______12 ______13 ______14 ______15

C: ______1 ______2 ______3 ______4 ______5 ______6 ______7 ______8 ______9 ______10 ______11 ______12 ______13 ______14 ______15

D: ______1 ______2 ______3 ______4 ______5 ______6 ______7 ______8 ______9 ______10 ______11 ______12 ______13 ______14 ______15

E: ______1 ______2 ______3 ______4 ______5 ______6 ______7 ______8 ______9 ______10 ______11 ______12 ______13 ______14 ______15

F: ______1 ______2 ______3 ______4 ______5 ______6 ______7 ______8 ______9 ______10 ______11 ______12 ______13 ______14 ______15

G: ______1 ______2 ______3 ______4 ______5 ______6 ______7 ______8 ______9 ______10 ______11 ______12 ______13 ______14 ______15

H: ______1 ______2 ______3 ______4 ______5 ______6 ______7 ______8 ______9 ______10 ______11 ______12 ______13 ______14 ______15

Bottom line Questionnaire for Muffin consumer Panel

Please answer these additional questions:

1. **Your Gender:** **Male/Female**
2. **Your Age group (yrs):** **<20** ○ **21-30** ○ **31-40** ○ **41-50** ○ **51-60** ○ **>60** ○
3. **Designation** **Student** ○ **Researcher** ○ **Teacher** ○
4. **Your Income Group (Rs.):** **<20,000** ○ **20,001-30,000** ○ **30,001-40,000** ○ **40,001-50,000** ○ **50,001-60,000** ○ **>60,000** ○
5. **Your Educational Level:** **B.Sc.** ○ **M.Phil.** ○ **Doctorate** ○ **Post Doctorate** ○
6. **Would you like to purchase these cardio protective (Omega-3 and Fiber enriched) Muffins if available in market?** **Yes** ○ **No** ○
